# Editorial: Unsupervised Learning Models for Unlabeled Genomic, Transcriptomic & Proteomic Data

**DOI:** 10.3389/fgene.2021.781698

**Published:** 2021-11-11

**Authors:** Jianing Xi, Zhenhua Yu

**Affiliations:** ^1^ School of Artificial Intelligence, Optics and Electronics (iOPEN), Northwestern Polytechnical University, Xi’an, China; ^2^ School of Information Engineering, Ningxia University, Yinchuan, China

**Keywords:** unsupervised learning, unlabeled data, OMICS data, genome, transcriptome, proteome

## Unsupervised Learning Models for Unlabeled Genomic, Transcriptomic and Proteomic Data

For unveiling the underlying biological mechanisms, the data of genomics, transcriptomics, proteomics, and other types of omics can offer informative cues for the understanding of underlying biological mechanisms (Muers, 2011). Since manual analysis of the huge amounts of these biological data is impractical, computational efforts of bioinformatics has been introduced as the key of unveiling the biological knowledge in omics data (Manzoni et al., 2018). A promising opportunity for omics data analysis is the recent developments in Artificial Intelligence (AI), which empowers bioinformatics research. Inspired by the advanced AI technology (Huang and Xi, 2020), a considerable number of effective and powerful intelligence approaches have been erupting in the bioinformatics research of omics data (Lightbody et al., 2019).

Nevertheless, it should be noted that, the paradigm of supervised learning framework are widely utilized in most of the recent emerging bioinformatics approaches (Min et al., 2017). Despite the achievements yielded by the existing omics data analysis, one of the main shortcomings is that these previously published approaches restrict annotated labels in the omic data as training set (Yu et al., 2019). In consideration of the massive amount of omic data involved in bioinformatics researches, there are extensively manual efforts required from experts, when such amounts of data are annotated with labels (Xi et al., 2021). Consequently, in omics data, a crucial bottleneck in bioinformatics research of omic data is the insufficiency of annotated labels (Yu et al., 2020).

For circumventing the shortage of manual annotations in omics data, a promising solution is to analyze the unlabeled omic data rather than labeled data, which can save considerable costs of annotation (Xi et al., 2020b). Instead of the widely used paradigm of supervised learning, introducing the paradigm of unsupervised learning can open a new window of omic research, demonstrating great potential for unlabeled omic data analysis Xi et al. (2020b). In comparison to the paradigm of supervised learning, unsupervised learning methods may throw light on the unlabeled omic data analysis, which can overcome the issue of high cost of annotated labels in omic data, and promote the research of omic data free from manual labels (Xi et al., 2020a).

This Research Topic focuses on the recent advanced approaches in the methodology of unsupervised learning and their applications on unlabeled omics data. A total of 9 articles related to unsupervised learning developments on the analysis of genomic data, transcriptomic data, proteomic data, and multi-omic data are included.

For genomic data analysis, three unsupervised learning approaches were published in the Research Topic, unveiling the aspects disease gene selection and copy number variation detection. Specifically, Xie et al. proposes a standard deviation and cosine similarity based unsupervised feature selection algorithms, which is capable of conducting gene selection for stable biomarkers of disease such as cancer through genomic data (Xie J. et al.). At the same time, Fan et al. proposes a hierarchical clustering based framework to predict the disease genes from stage-specific gene regulatory networks (Fan et al.). Furthermore, Xie et al. proposes a local density and minimum distance based density peak clustering method called dpCNV, for detecting relative large range copy number variation from DNA sequencing data (Xie K. et al.). These advanced approaches mainly cover the methodology of feature selection, hierarchical clustering, and density peak estimation, expanding the frontiers of genomic researches.

For transcriptomic data analysis, there are two papers contributing to RNA data research as the roles of bioinformatics tools. One research in this Research Topic is focusing on in single-cell RNA sequencing (Yu et al., 2021), which aims to overcome the zero-inflated data caused by dropout events (Zhao et al.), where Zhao et al. proposes a dimensionality reduction approach on single-cell RNA sequencing data, which is based on a hierarchical autoencoder consisting of a deep count autoencoder for denoising and a graph autoencoder for dimensional reducing. Meanwhile, for long intergenic non-coding RNA (lincRNA) analysis, Lin and Ma proposes a non-negative matrix factorization approach with co-regularization to predict disease-lincRNA associations (Lin and Ma), which integrates four types of information associated to lincRNA. Generally, the two researches are concentrating on the advanced frontiers of either AI technology research or transcriptomic research.

For proteomic data analysis, there are two articles offering the unsupervised learning methods on two aspects. One aspect is to detect overlapping structures in protein functional modules from proteomic data of protein-protein interactions, where Wang et al. proposes a neighboring local clustering coefficient based overlapping community detection algorithm to mine functional modules in these interactions (Wang Y. et al.). Another aspect is to measure the similarity of proteins, where Zhang et al. further incorporates structural information of Gene Ontology (GO) graph to compensate the consideration of only information content of GO terms, and calculates the similarity of proteins through graph embedding methods (Zhang et al.). These protein interaction graph based approaches in the Research Topic also illustrate the frontiers of proteomic research.

For multi-omic data analysis, this Research Topic also collected two studies which include more than one type of omic data. Detailly, Wang et al. proposes a joint matrix tri-factorization framework for discovering complex biological processes (CBPs) of multi-omics molecules regulation, which reflect the activities of various molecules in living organisms (Wang B. et al.). Moreover, in the prediction of cancer subtypes, to effectively utilize rich heterogeneous information in the multiple view fusion graph of multiple omics data, Liu et al. proposes a multi-smooth representation fusion based multi-view spectral clustering method, which consists of graph construction, graph fusion, and spectral clustering for clustering of cancer subtypes from multi-omic data (Liu et al.). These works also show the frontiers of multi-omic research.

In brief, This collection of contributions in the Research Topic provide a window into the frontiers of unsupervised learning models for unlabeled genomic, transcriptomic and proteomic data. Given the remarkable success of unsupervised learning application in bioinformatics problems, we hope that these approaches can throw light on the problem of data annotation cost, extending the frontiers of bioinformatics research of omic data.
